# Consensus building to improve the physical health of people with severe mental illness: a qualitative outcome mapping study

**DOI:** 10.1186/s12913-015-0744-0

**Published:** 2015-03-03

**Authors:** Carolyn Ehrlich, Elizabeth Kendall, Nicolette Frey, Michelle Denton, Steve Kisely

**Affiliations:** Centre of National Research on Disability and Rehabilitation, Menzies Health Institute, Queensland, Griffith University, Logan Campus, Meadowbrook, QLD 4131 Australia; School of Medicine, University of Queensland, St Lucia Campus, Brisbane, QLD 4101 Australia

**Keywords:** Nominal group technique, Integration, Interdependent action, Wicked issues, Action pathways, Planning, Generating solutions

## Abstract

**Background:**

The poor physical health of people with severe mental illness (SMI) is often attributed to lifestyle, disease-related medication side effects and disparate provision of healthcare. The complexity and inexact nature of this issue prohibits the identification of a clear and concise causal pathway, which in turn leads to uncertainty and imprecision about the most appropriate action to address the problem. One proposed solution is to integrate care across multiple organisations and sectors through collaborative processes. The objective of this study was to identify collective pathways of action that were consensually developed and which could be initiated by clinicians to improve the physical health of people with severe mental illness.

**Methods:**

Eighteen participants from a service catchment area in Australia were involved in a consensus-building workshop. This resulted in participants identifying and committing to a range of collaborative actions and processes to improve the physical health of people with severe mental illness. Consensus building was combined with an outcome mapping process, which has previously been used to facilitate health system integration. Data from the consensus-building workshop were thematically analysed and used to create an outcome map.

**Results:**

Participants identified that accessible, continuous, holistic, consumer-driven, recovery-oriented care was required if improved physical health of people with SMI were to be achieved. However, this all-encompassing care was dependant on a wide-ranging philosophical shift in two areas, namely societal stigma and the dominance of pharmacological approaches to care. Participants believed that this shift was contingent on the attitude and behaviours of healthcare professionals and would require an inclusive, networked approach to care delivery and maximal utilization of existing funding.

**Conclusions:**

Rarely do multiple stakeholders from different sectors within the healthcare system have the opportunity to come together and create a collective vision for improving the health of a specific population in a defined area. We used a consensus building approach to generate solutions, actions and goal statements, which were then used to create a visual map that provided a purpose and signposts for action, thereby maximising the potential for cohesive action across sectors.

## Background

*“…social problems are not just “complex problems”… it is their openness that makes them elusive”* ([1], p.279).

The poor physical health of people living with severe mental illness (SMI) has been reported for more than 70 years [2,3] and it is well established that that there is an increased risk of death up to five times that of the non-mentally ill population. This results in 25–30 years of life lost, primarily as a result of chronic physical illness [4-6]. In Australia, people with SMI have a life expectancy up to 30 years less than the remainder of the population [7,8], and explanations for this disparity frequently focus on lifestyle factors such as obesity, lack of exercise, and alcohol and tobacco use. Despite overall population mortality gains, health outcome disparity between people with and without mental illness is increasing because the excess mortality experienced by people with mental illness remains elevated [9]. Taking local action to address this issue features prominently in current Australian mental health policy [10]. However, contemporary mental healthcare is complex [11], and the contributing factors for poor physical health and increased mortality are diverse and multi-faceted.

Lifestyle, disease-related medication side effects and disparate provision of healthcare inter-relate in ways that contribute to poor health and increased mortality of people with SMI [7]. The complexity and inexact nature of these contributing factors create problems that can be described as ‘wicked’ [12]. Wicked problems are socially complex, rarely stable, and symptomatic of other equally complex problems [12,13]. Consequently, they are not easily solved and actions to address one issue often leads to unforeseen consequences [12,13]. Therefore, a clear and concise causal pathway is elusive, which in turn leads to uncertainty and imprecision about the most appropriate action required to address the problem at hand. Furthermore, responsibility for responding to wicked problems rarely resides within the remit of one organisation or one level of government [13]. Thus, one proposed solution to addressing wicked issues such as that of improving the physical health outcomes of people with SMI is to integrate care across multiple organisations and sectors [7,14].

Integration is required if common goals are to be successfully achieved within complex systems [15]. Consequently, collaborative rather than competitive or authoritative approaches are necessary [16]. These collaborative approaches require acknowledgment of, and interaction with, the social processes that link and bind system components into large and interconnected networks [17]. However, collaborative processes are time consuming and significant effort is required to communicate and achieve consensus [16]. Therefore, if integration is to be achieved, we need to find effective collaborative processes that expeditiously support communication and build consensus amongst stakeholders.

Outcome mapping is one recent approach that has been used to facilitate health system integration and is suited to working in situations where multiple stakeholders are required to practice collaboratively [18]. Tsasis and his colleagues [18] recommend that outcome mapping is beneficial for (1) identifying and linking outcomes with actions, (2) making sense of complex systems, (3) building collaborative capital, (4) exploring the boundaries, gaps and links between social networks across the care continuum, and (5) identifying specific organisational and professional roles that influence outcome achievement. Therefore, we reasoned that outcome mapping would potentially facilitate action which was collaborative and cross-sectoral, and which would ultimately improve the physical health of people with SMI.

In situations that are required to deal with uncertain, complex or controversial issues [19], a consensus building process maximises participants’ collective expertise and produces conceptual frameworks that are more likely to be accepted in practice [20,21]. Therefore, we combined outcome mapping with a consensus building approach and explored the actions, processes and high level outcomes that stakeholders across government, non-government and primary healthcare sectors believed were essential for improving the physical health of people with SMI. This paper reports the use of this combined process, which was one part of a larger research project. The objective of this component of the larger study was to identify collective pathways of action that were consensually developed and which could be initiated by clinicians to improve the physical health of people with severe mental illness. Ethics approval was received from the relevant University and Queensland Health Human Research Ethics Committees.

## Method

### Participants

Eighteen participants from a geographically defined catchment area in Queensland, Australia were involved in a consensus-building workshop. Eleven participants were from community based public mental health services, two from general practice, and five from two different non-government organisations (NGOs). All participants were actively engaged in providing healthcare services to people with a SMI, and their roles ranged from support worker responsibilities to those with organisational management commitments.

### Consensus-building workshop

A consensus-building workshop was held in May 2013 to: (1) generate potential solutions to address the issue of improving the physical health of people with a SMI, and (2) prioritize the solutions. Although multiple consensus building processes are available [22], we used the nominal group technique which has been used extensively in health research [23,24]. Based on this technique, the workshop was structured in four parts. First, researchers provided participants with an overview of evidence to date [22], which included a summary of the relevant literature and a synopsis of the results from previous data collection (i.e., a health promotion survey and initial qualitative interviews). Second, participants were asked to individually generate potential solutions to the problem of improving the physical health of people with a severe mental illness. One by one, each participant was given the opportunity to inform the group of one of their solutions. The process was continued until all generated solutions had been captured. At the same time as participants were nominating solutions; researchers were scribing potential solutions onto large sheets of paper, which were then grouped to reflect similar themes and displayed on the walls of the room in which the workshop was being held.

Third, each participant was provided with a total of fifteen adhesive dots and instructed to place five dots against their most important solution, four against the next most important and so on. Thus, participants ranked a total of five possible solutions in order of importance. Once this exercise was completed, participants and researchers were able to ascertain the most highly important solutions for the entire group. The dots were colour coded according to sector, which made it possible to ensure that the most highly valued solutions represented a solution for all sectors. Once the solutions had been generated and prioritized, participants individually completed the task of identifying actions they could take to achieve the priority solutions. Participants then shared these actions with the group. In the final component of the workshop, participants individually committed to a path of action.

### Data analysis

The entire workshop was recorded and transcribed verbatim. Data were thematically analysed [25] and then used in an outcome mapping process [18]. Several sources of data were used to triangulate the analysis process [26]. First, the worksheets on which participants had recorded their solutions and practices were reviewed. Second, the transcripts of participant feedback to the group were analysed to ensure that detailed meaning of the content of the written worksheets was accurately reflected in the analysis. Third, the preliminary themes identified by researchers during the feedback sessions were re-visited to ensure that they were an accurate reflection of the solutions presented by participants.

**Figure 1:**
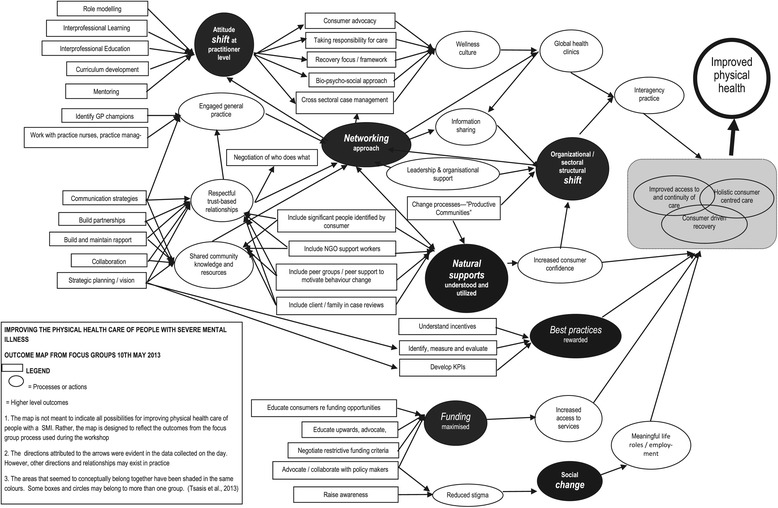
**Cross sectoral roadmap to improved physical health outcomes for people with severe mental illness.**

Constant comparison of all three sources of data occurred throughout analysis [27]. Findings from the analysis were then categorized into either: (1) actions and processes, or (2) higher level outcomes [18]. Finally, an outcome map was drawn to reflect the pathways or linkages to show how identified actions and processes would lead to the desired outcomes [18] (see Figure 1). The outcome map was circulated via electronic mail and in hard copy to all participants, and feedback was invited. Participants were then contacted by telephone to ascertain further feedback. All participants agreed that the resultant map accurately reflected the consensus that was built during the workshop.

## Results

Overwhelmingly, participants identified the need to take collective action to improve the physical health of people with SMI by ensuring care continuity and by adopting holistic and individually-driven recovery oriented approaches across sectors. Our thematic analysis revealed that, if these goals were to be achieved, several changes to care provision approaches were required. First, there needed to be a wide-ranging philosophical shift by practitioners, organisations and communities. Second, people with SMI needed to be included in care choices that were provided within supportive networks. Third, wherever possible, funding structures needed to be maximised. Finally, the attitudes of practitioners and the way in which best practices were understood and rewarded needed to be addressed. By using a consensus-building approach, participants were able to identify ways in which each of these goals could potentially be realised through collaborative action.

Twenty-two prospective solutions were proposed by participants, and were broadly categorized into four solution categories, namely (1) *collaboration and communication*, (2) *education*, (3) *better funding mechanisms,* and (4) *a wellness attitude*. Collaboration and communication was most highly prioritized by participants (39% of total number of votes), followed by a wellness attitude (27% of total number of votes), better funding mechanisms (18% of total number of votes), and finally, education (16% of total number of votes). However, when reviewed by sector, the non-government sector placed a higher priority on adopting a wellness attitude than on collaboration and communication (refer Table 1).

**Table 1:** **Prioritization of Each Broadly Themed Solution to Improving the Physical Health of People with a Severe Mental Illness**

	**Overall priority (%)**	**Private sector (%)**	**Public sector (%)**	**Non-government sector (%)**
**Collaboration and communication**	39	37	44	28
**Education**	16	29	12	20
**Better funding mechanisms**	18	30	17	18
**Wellness attitude**	27	4	27	34*
**Total**	100	100	100	100

*Wellness attitude rated more highly than collaboration and communication in the non-government sector.

Once the prioritised solutions were combined with proposed actions during the post-workshop analysis, some consistent pathways emerged. Participants believed that physical health would be improved by working towards improved access to, and continuity of, care *“…so we’re working with the NGO and the primary health provider, doing it together, then hopefully, one day, we will discharge them and it will continue the same”*; holistic care *“…to be able to offer a holistic solution for people…”* and individual-driven recovery *“…We come to them with opportunity for change… We actually don’t have the right to tell people how they live”* (refer Figure 1). Although all pathways were believed to be important, achieving an extensive philosophical shift was required. Participants identified two approaches which, if adopted, had the potential to achieve the desired shifts: (1) active inclusion of people with SMI within supportive networks, and (2) maximising the use of existing funding. The moderating factor on which philosophical changes hinged was the attitude and behaviours of professionals.

### Wide-ranging philosophical shift

Participants discussed the need to achieve a wide-ranging shift in community, practitioner and agency behaviours and attitudes. There were two important areas in which these philosophical shifts were required. First the stigma associated with having a mental illness and the perpetuation of stigma at multiple levels needed to be addressed *“…because you know stigma and prejudice influence the way that you even just talk to people…”*. Therefore:

*One of the big challenges I think we face at the moment is raising awareness and challenging community understanding and attitudes that influence policy, that influence our practice every single day. I think it’s the biggest challenge that as mental health practitioners we face because everyone brings an attitude into their work….*

Secondly, participants were concerned about the dominance of pharmacological approaches to care. Achieving recovery driven and continuous care was believed to be dependent on health professionals adopting a holistic rather than solely biomedical approach:

*It’s about creating worth and meaning for people and I think that can get lost in the discourse about ‘how are they being treated’ and ‘what are their medications’?… I am aversive to it dominating everything we think about, only because I know from working with people that even though they are ill, it’s not always about the medical treatment. It’s about the quality of their life, and whether or not their children are actually getting the education they need, and whether or not their partner is going to get pulled up by the cops [police] again next week… Then what’s he going to do for his income, because he can’t go to work. That’s what their lives are about.*

### Adopting an inclusive approach within supportive networks

Participants wanted to ensure that people with SMI were offered choices, that their choice was respected, and that they were afforded the opportunity to change their mind:

*…consumer choice and respecting a person’s right… to make the choices that we don’t necessarily agree with… but after offering them education if they make that choice long term to not just accept it long term. That it [the choice the consumer made] be re-visited at an agreed upon time to see if they have changed their mind….*

Therefore, relationships with consumers needed to be inclusive and focus on their hopes, goals and dreams rather than on provider or health service oriented goals. Many participants believed that the provision of physical healthcare was unlikely to be optimal when consumers and significant others were not included in care decisions. For instance, one participant stated *“…if you make it [care processes] clinical all the time, then… it becomes about what the clinicians need… not so much about what the person needs…”.* Participants also believed that the involvement of peer groups and peer support workers would motivate people with SMI towards healthy lifestyle changes. Involving individuals and their natural supports in care was believed to enhance their confidence and assist engagement with health professionals and healthcare agencies.

A network approach was the key to achieving inclusiveness and collaborative practice. Such an approach was reliant on *“…knowing what you’ve got in other teams and what other expertise you can link into”.* However, this required the following: an engaged general practice sector; respectful, trust-based relationships across all sectors; and shared community knowledge and resources. A network approach was therefore based on rapport and partnership building, collaboration and communication strategies, and a shared vision. An interprofessional collaborative approach would lead to greater engagement of general practice and integration of care provision across professions, groups and sectors. However, such a process was not easy, or quick, and required effective leadership:

*I’m part of the … group that meet every Monday… there’s a lot of relationship building that happens within that meeting. It wasn’t always this way. This group got together and it had teething problems, communication problems. It took a long time and a good chairman to pull that group together for it to work… When I think about them today, there have been relationships built… from many agencies, sharing of resources and also information sharing. That’s worked well.*

Many participants recognised that adopting a network approach would require a structural shift in the way that organisations and sectors currently practice. Such a shift was dependent on organisational support and proactive, effective leadership and “*…clear expectation[s] as to performance and outcomes in terms of collaboration amongst services and with service users to achieve physical care outcomes…”.*

### Maximising funding structures

Despite the importance of addressing stigma, access to funding was sometimes reliant on accepting a potentially stigmatising label, thereby perpetuating stigmatisation:

*…I’m incredibly uncomfortable with the concept of a disability, but I’m also very aware that there’s a monstrous great bucket with billions of dollars in it that sits under that label of disability, or is about to… if you deny the fact that things are [defined as a] disability, you cannot have funding. So you’ve got to collaborate with a somewhat stigmatising label. I guess, at the end of the day, if what you want to do is get some funding in to help some people, we probably have to compromise on our language…*

Thus, practitioners and consumers faced a dilemma because *“Dollars make a difference to everything that the carers and consumer that I’ve worked with are able to do”.*

Participants also agreed that funding was a major barrier to improved access and continuity of care. Although it was essential that *“You have your GP there [at case conferences] [because the GP] really is the central person”,* engaging general practitioners in collaborative and inclusive case reviews was restricted by existing funding models:

*“There is a case management [Medicare reimbursement] number, but it’s not very much and the GP only has to be there for 15 minutes… it’s not really conducive to primary care being involved… [and] the consumer actually doesn’t have to be there for the GP to claim the item number [payment from Medicare]”.*

Recognising that broad funding structures were unlikely to be changed in the short-term, it was believed that advocacy and collaboration were seen as important initial steps. For instance, at the local level, people with SMI could be educated about existing funding opportunities and information shared about future opportunities. The use of Medicare Benefits Scheme (MBS) items could also be maximised, and community knowledge about available resources enhanced.

### Practitioner attitude

The attitude of practitioners clearly affected the way in which they took responsibility for care, understood and engaged with a recovery focus and cross sectoral case management and evaluated the outcomes of care they provided. Participants believed that a change in the behaviour and actions of some health professionals would be required to achieve improved physical health outcomes of people with SMI:

*…attitudes and attitudinal shift… that’s the missing part, isn’t it? You, out of your professional responsibility choose to do that [good practice]. That’s great, but there’s nothing embedded to make sure that others follow that line… It’s the attitude…*

A behavioural shift was more likely to occur when inclusive ways of working were modelled and professionals able to learn from each other in the workplace. Although inter-professional learning underpinned behavioural and attitudinal change, clinical specialisation should be preserved and valued:

*So, we’ve got nurses doing social work and social workers doing medicine and it – there’s certainly a lot of common ground and I’m all for that, but it’s – we’ve kind of sacrificed speciality…*

Additionally, education curricula that supported a change in professional attitudes and behaviours should be developed in conjunction with, and endorsed by, peak professional bodies:

*…raises the issue that the professional organisations… I think as professional organisations we have to lift our game… I think we can take a leaf from the College of Psychiatrists, who have addressed this [physical healthcare] now in examination criteria. So, you don’t get to be a psychiatrist unless you can demonstrate that you’re able to look at these issues and deal with them…*

Closely linked with a move away from a solely biomedical approach, was the adoption of a wellness culture which was described as the integration of advocacy, health professional responsibility, a recovery focus, a bio-psycho-social approach and cross sectoral case management at the very least:

*So essentially moving away from a reactive therapeutic sort of focus or dominance at least to beyond preventative even; way more upstream in terms of a wellness focus where we have a perspective in terms of the populations, so a macro-perspective in terms of population and what we contribute to a sense of wellbeing or wellness…, but nobody really knows what that wellbeing bit means.*

Multi-disciplinary, multi-agency health clinics were one approach where a wellness culture could be fostered.

### Understanding and rewarding best practices

Participants believed that for behaviour and attitude change to be achieved, practice needed to be monitored, and best practices rewarded:

*…we know that people change their practice… so you want to reward certain behaviours and you want to ensure that those behaviours [have] consistent acceptance by clinical teams…*

For some participants, this meant that key performance indicators needed to be developed so that the impact of collaboration could be measured. Thus, it was essential that incentives for behaviour were understood, and that the activities and processes necessary to create the best possible outcomes for people with mental illness were identified, measured and evaluated:

*…So, I think we can be specific about… what are we trying to reward and therefore to increase? The focus on identification… of physical health problems or identification of opportunities for prevention with regard to physical health problems… nicotine use obviously is very widespread, but we rarely actually make the diagnosis… we should be able to diagnose nicotine users…*

In summary, participants involved in a consensus-building workshop identified several pathways of action that, when enacted would contribute towards improving the physical health of people with SMI. Although each identified pathway of action contributed to the desired outcomes, a consumer driven recovery was most closely aligned with understanding and utilizing natural supports and acting to reduce the stigma of mental illness. A consumer driven recovery coexisted with holistic consumer centred care, which was dependent on a move away from the dominant medical discourse that traditionally surrounded care provision towards an inclusive approach that enabled practitioners to adopt a wellness culture. Sectors also needed to shift towards collaborative action to ensure that consumer identified needs were adequately met by the professional or team or agency with the most appropriate resources and skills. Although a consumer oriented recovery approach was ideal, participants expressed frustration about system-imposed processes that were particularly problematic for care continuity when consumers needed to reengage with services:

*…with reengagement… should you wish to get back in contact with the service, you’ve then got to climb this weary ladder of acute care, emergency department, mental status examination, the whole box and dice, just to reengage with the clinician you saw last week. So the GPs also perceive this…If they’ve [consumers] moved out of services and they [consumer] need to get back in, it’s as long as somebody is in there advocating for them, that’s the thing that’s the most important. I don’t know how well that happens…*

Improved access to, and continuity of, care was reliant on funding maximisation and appropriate sharing of information and resources throughout the network of professions, teams and agencies that supported the consumer.

## Discussion

We used a consensus building process to actively assist government, non-government and primary care stakeholders explore different perceptions about existing problems and their solutions; and to jointly develop and commit to strategies for interdependent action [18,22,28]. The most important strategy identified by participants in our study was the need for extensive changes in culture and values (i.e., philosophical shifts), which would ultimately be facilitated by engaging in inclusive approaches to care, networks of action and maximising funding opportunities. However, the intersection between mental and physical healthcare was frequently associated with conflicted values and interests, which contributed to complex divisions of work between individuals, teams and organisations; and made the formulation of problems and proposed solutions contestable [11,17]. Thus, the attitudes and behaviours of individual practitioners were instrumental factors that influenced the way that inclusivity and networking were approached.

Engaging people with SMI and other significant people within their social networks in the provision of care is challenging. In response, user involvement and recovery-oriented practice features prominently in contemporary mental health policy and literature [29,30]. Recovery-oriented practice requires organisational commitment, supportive working relationships, a willingness to promote citizenship, and practitioner support directed at achieving a personally defined recovery [29]. Thus, integrating recovery-oriented practice into everyday mental health care will assist organisations and professionals to achieve many of the outcomes that were developed consensually and presented in this paper.

Unsurprisingly, participants attributed a high level of importance to communication, sharing community knowledge and resources, developing respectful trust-based relationships and negotiating with other stakeholders. Thus, networks and networking were essential. However, if the desired philosophical shifts were to be attained, it was essential to build the networks’ capacity for collaboration so that the transfer, receipt and integration of knowledge between individuals could be effectively managed [31]. The consensus building process we used contributed to knowledge transfer, receipt and integration. However, the bigger future challenge will be sustainable and collaborative approaches that include not only professional knowledge, but also the critical knowledge that resides within the social and extended networks of people with SMI.

No single organisation, team, disciplinary group or individual practitioner had the entire suite of knowledge and skills to deliver all components of the identified strategies. Therefore, integrated solutions that facilitated program and service coordination needed to be developed [32]. In response, we created an outcome map to capture both the higher level outcomes that participants were striving to achieve and the processes and actions that had been agreed by participant stakeholders [18]. The outcome map visually represented both the socially defined meaning of the problem of the poor physical health of people with a severe mental illness and the multiple action pathways required [18,32].

The consensus building approach we used in our study, combined with the consequent outcome map, was consistent with approaches to deal with “wicked” issues. Although there was no definite way to formulate either the problem or an absolute solution [17], we have been able to achieve consensus about the breadth of the problem and an indication of a potential pathway. By representing the pathway forward as an outcome map, participants had visual access to multifaceted action pathways, which assisted them to identify where and how they were able to act individually and collectively to improve the physical health of people with SMI. However, even though we have developed a map that contextualizes mutual cooperation between organisations, planning and delivering the actions required to achieve the desired outcomes is likely to be challenging [33].

First, funding is often a powerful incentive that underpins and drives action [34]. Second, even when robust solutions are developed, aligning action at the level of individual employees is difficult [34]. Participants recognised that identifying appropriate performance indicators were key to achieving desired behaviour changes. However, not all change leads to improvement [35]. Therefore, appropriate performance measures would need to be identified because when multifaceted interventions were grounded in an accurate assessment of barriers to change, successful behaviour change was more likely to result [36]. There are several theories and frameworks that were likely to facilitate understanding and mitigate implementation barriers and the adoption of new behaviours in practice including general implementation theory [37]; diffusion of innovation in service systems [38]; and organisational readiness to change theory [39]. Therefore, further research is required using some or all of these theories to test the action strategies identified in the outcome map.

There are several limitations that can be identified in this study. First, defining the problems and issues associated with complicated issues requires all elements of the system to be involved in a learning based problem solving process [16]. However, although we have engaged people with SMI and their carers in separate data collection processes that aimed to explore their experience of receiving physical health care and which will be reported elsewhere, they were not involved in this component of the research. This may have limited the accuracy of information on which consensus development and solution generation was based. Additionally, even though participants identified the need to work across sectors, participants from other important sectors such as law enforcement, housing and employment were not included in the consensus development process. Although including these sectors would have been beneficial, it was beyond the scope of this project and should be considered in future research that is seeking to address complex issues. A further limitation to this research is that the research was conducted in a defined geographical area and might therefore, not be transferrable to other contexts.

## Conclusions

The main outcome from this research was to create a visual map that provided signposts for collective action across health and social care sectors. It is rare for different sectors within these systems to have the opportunity to collectively create a vision for improving the health of a specific population in a defined area. We used a consensus building approach to generate collective solutions to improving the physical health of people with SMI. The resultant outcome map provides sufficient detail for each individual, team or organisation to take action that, when added with the action of other individuals, teams and organisations has a better chance of meeting the goal of improving the physical health of this population. However, multiple challenges remain. The approach that we have taken is one of many incremental steps that will be required to lessen the health disparity experienced by this population.

## Abbreviations

GP: General practitioner
SMI: Severe mental illness
MBS: Medicare benefits scheme
NGO’s: Non-government organisations
